# CD4^+^ T Cells Expressing PD-1, TIGIT and LAG-3 Contribute to HIV Persistence during ART

**DOI:** 10.1371/journal.ppat.1005761

**Published:** 2016-07-14

**Authors:** Rémi Fromentin, Wendy Bakeman, Mariam B. Lawani, Gabriela Khoury, Wendy Hartogensis, Sandrina DaFonseca, Marisela Killian, Lorrie Epling, Rebecca Hoh, Elizabeth Sinclair, Frederick M. Hecht, Peter Bacchetti, Steven G. Deeks, Sharon R. Lewin, Rafick-Pierre Sékaly, Nicolas Chomont

**Affiliations:** 1 Centre de Recherche du Centre Hospitalier de l’Université de Montréal, Montreal, Quebec, Canada; 2 Vaccine and Gene Therapy Institute Florida, Port St Lucie, Florida, United States of America; 3 Department of Infectious Diseases, Monash University and Alfred Hospital, Melbourne, Victoria, Australia; 4 The Peter Doherty Institute for Infection and Immunity, University of Melbourne, Victoria, Australia; 5 Department of Epidemiology and Biostatistics, University of California, San Francisco, California, United States of America; 6 Department of Medicine, University of California, San Francisco, California, United States of America; 7 Case Western Reserve University, Cleveland, Ohio, United States of America; 8 Department of Microbiology, Infectiology, and Immunology, Université de Montréal, Faculty of Medicine, Montreal, Quebec, Canada; Vaccine Research Center, UNITED STATES

## Abstract

HIV persists in a small pool of latently infected cells despite antiretroviral therapy (ART). Identifying cellular markers expressed at the surface of these cells may lead to novel therapeutic strategies to reduce the size of the HIV reservoir. We hypothesized that CD4^+^ T cells expressing immune checkpoint molecules would be enriched in HIV-infected cells in individuals receiving suppressive ART. Expression levels of 7 immune checkpoint molecules (PD-1, CTLA-4, LAG-3, TIGIT, TIM-3, CD160 and 2B4) as well as 4 markers of HIV persistence (integrated and total HIV DNA, 2-LTR circles and cell-associated unspliced HIV RNA) were measured in PBMCs from 48 virally suppressed individuals. Using negative binomial regression models, we identified PD-1, TIGIT and LAG-3 as immune checkpoint molecules positively associated with the frequency of CD4^+^ T cells harboring integrated HIV DNA. The frequency of CD4^+^ T cells co-expressing PD-1, TIGIT and LAG-3 independently predicted the frequency of cells harboring integrated HIV DNA. Quantification of HIV genomes in highly purified cell subsets from blood further revealed that expressions of PD-1, TIGIT and LAG-3 were associated with HIV-infected cells in distinct memory CD4^+^ T cell subsets. CD4^+^ T cells co-expressing the three markers were highly enriched for integrated viral genomes (median of 8.2 fold compared to total CD4^+^ T cells). Importantly, most cells carrying inducible HIV genomes expressed at least one of these markers (median contribution of cells expressing LAG-3, PD-1 or TIGIT to the inducible reservoir = 76%). Our data provide evidence that CD4^+^ T cells expressing PD-1, TIGIT and LAG-3 alone or in combination are enriched for persistent HIV during ART and suggest that immune checkpoint blockers directed against these receptors may represent valuable tools to target latently infected cells in virally suppressed individuals.

## Author Summary

The persistence of HIV in a small pool of long-lived latently infected resting CD4^+^ T cells is a major barrier to viral eradication. Identifying cellular markers that are preferentially expressed at the surface of latently infected cells may lead to novel therapeutic strategies to cure HIV infection. We identified PD-1, TIGIT and LAG-3 as markers preferentially expressed at the surface of infected cells in individuals receiving ART. CD4^+^ T cells co-expressing these markers were highly enriched for cells carrying HIV. Our results suggest that PD-1, TIGIT and LAG-3 may represent new molecular targets to interfere with HIV persistence during ART.

## Introduction

Although antiretroviral therapy (ART) is highly effective at suppressing HIV replication, viral reservoirs persist despite treatment and lead to rapid viral rebound when ART is interrupted [1–4]. A major step to achieve natural control of HIV replication after ART cessation would be to eliminate, or at least reduce, the number of long-lived infected cells from which HIV reignite infection. The characterization of cell surface markers that could identify HIV-infected cells persisting during ART is a research priority towards an HIV cure [5] as it could lead to the development of novel eradication strategies.

Several subsets of CD4^+^ T cells harbor replication-competent HIV during ART. These CD4^+^ T cells are usually defined on the basis of their differentiation stage [6–8], functionality or homing potential [9,10]. Central memory (T_CM_) and transitional memory (T_TM_) CD4^+^ T cells were identified as the major cellular reservoirs for HIV during ART [6]. More recently, a less differentiated subset of long-lived cells with high self-renewal capacity, the stem-cell memory CD4^+^ T cells (T_SCM_), has been identified as a main contributor to long-term HIV persistence [7,8]. The functional and homing capacities of CD4^+^ T cells also dictate their capacity to serve as persistent reservoirs for HIV: Th17 and Th1/Th17 CD4^+^ T cells as well as cells expressing CCR6 and CXCR3 show increasing contribution to the viral reservoir with duration of ART [11,12].

Immune checkpoint molecules (ICs) are co-inhibitory receptors which down-modulate immune responses to prevent hyper-immune activation, minimize collateral damage, and maintain peripheral self-tolerance [13]. ICs are up regulated upon T-cell activation and constrain the effector response through feedback inhibition. Overexpression of these molecules is associated with T-cell exhaustion and dysfunction in cancer and chronic viral infections, including HIV [14–17]. We hypothesized that ICs, through their ability to inhibit T-cell activation, will favour HIV latency during ART, and that CD4^+^ T cells expressing ICs would be enriched for persistent HIV in individuals receiving ART.

We focused our analysis on 7 ICs, namely PD-1 (programmed cell death-1), CTLA-4 (cytotoxic T-lymphocyte-associated protein 4), LAG-3 (lymphocyte activation gene 3), TIGIT (T-cell immunoglobulin and ITIM domain), TIM-3 (T cell immunoglobulin and mucin 3), CD160 and 2B4 (CD244).

PD-1, a member of the B7-CD28 superfamily, enforces an inhibitory program that blocks further TCR-induced T-cell proliferation and cytokine production [18,19]. In HIV infection, high levels of PD-1 are associated with T cell exhaustion [14–16,20] and incomplete immunological response to ART [21]. CTLA-4, a CD28 homolog, regulates the amplitude of T-cell activation by both outcompeting CD28 in binding CD80 and CD86, as well as actively delivering inhibitory signals to T cells [13]. TIGIT, which also belongs to the B7/CD28 superfamily, acts as a co-inhibitory molecule by directly down regulating proliferation of human T cells [22], but also by modulating cytokine secretion of DCs, decreasing IL-12 and enhancing IL-10 productions [23]. TIGIT has been recently associated with CD8^+^ T-cell dysfunction during HIV infection [24]. The expression of 2B4 (CD244), a member of the signalling lymphocyte activation molecule (SLAM) is also modulated on T cells during HIV infection [17,25]. LAG-3, a member of the immunoglobulin superfamily, is structurally highly homologous to the CD4 receptor and share MHC-II as a ligand [26]. Its expression on T regulatory cells plays a role in the modulation of T cell homeostasis and effector T cell responses [27,28]. TIM-3 is also an immunoglobulin superfamily member and its expression is increased on HIV-specific CD8^+^ and CD4^+^ T cells [29,30]. Finally, CD160, through its binding to its ligand Herpes Virus Entry Mediator (HVEM), an atypical member of TNF-receptor superfamily, delivers a co-inhibitory signalling to CD4^+^ T cells or CD8^+^ T cells dampening their activation in HIV-infected individuals [31,32].

To assess the relationship between the expression of these ICs and HIV persistence, we analysed the association between their levels of expression on CD4^+^ T cells and the size of the HIV reservoir in individuals receiving ART for at least 3 years.

## Materials and Methods

### Study participants

Forty-eight HIV-infected participants receiving suppressive ART were recruited at the University of California San Francisco (UCSF) for this cross-sectional study. Participants were receiving ART for >3 years, had CD4^+^ T-cell count >350 cells/μl and HIV RNA <40 copies/mL as measured by the Abbott real time HIV-1 PCR for at least 3 years. Whole blood (50mL) was collected by regular blood draw. For cell sorting experiments, 27 HIV-infected individuals were enrolled at UCSF and at VGTIFL and underwent leukapheresis.

### Ethics statement

All subjects signed informed consent forms approved by the UCSF and Martin Memorial Health Systems review boards (IRB #10–1320, Ref # 068192 and FWA #00004139, respectively).

### Immunophenotyping

PBMCs were isolated from peripheral blood and leukapheresis using previously described methods [6,33]. Cryopreserved PBMCs were thawed, washed and stained for phenotyping or cell sorting. Two antibody panels were used to measure the expression of IC in subsets of memory CD4+ T cells. The same antibody backbone was used in the two panels: CD3-Alexa700 (clone UCHT1, BD#557943), CD4-QDot605 (clone S3.5, Invitrogen#Q10008), CD8-PB (clone RPA-T8, BD#558207), CD14-V500 (clone M5E2, BD#561391), CD19-AmCyan (clone SJ25C1, BD#339190), LIVE/DEAD Aqua marker (Invitrogen#L34957), CD45RA-APC-H7 (clone HI100, BD#560674), CD27-BV650 (clone O323, Biolegend#302828) and CCR7-PE-Cy7 (clone 3D12, BD#557648). The following antibodies were added to this backbone: PD-1-AF647 (clone EH12.1, BD#560838), CTLA-4-PE (clone BNI3, BD#555853), LAG-3-FITC (R&D#FAB2319F), TIGIT-PerCP-eF710 (clone MBSA43, eBioscience#46-9500-41), TIM-3-PE (clone F38-2E2, Biolegend#345006), CD160-AF488 (clone By55, eBioscience#53–1609), 2B4-PerCP-Cy5.5 (clone C1.7, Biolegend#329515). For expression of all ICs, gates were defined using fluorescence minus one controls. CD4^+^ T-cell subsets were identified by CD27, CD45RA, and CCR7 expression on CD4^+^ T cells after exclusion of dump positive cells (LIVE/DEAD, CD14 and CD19). ICs were measured in gated CD4^+^ T-cell subsets including naïve CD4^+^ T cells (CD3+CD8-CD4+CD45RA+CCR7+CD27+), central memory CD4^+^ T cells (CD3+CD8-CD4+CD45RA-CCR7+CD27+), transitional memory CD4^+^ T cells (CD3+CD8-CD4+CD45RA-CCR7-CD27+), effector memory CD4^+^ T cells (CD3+CD8-CD4+CD45RA-CCR7-CD27-) and terminally differentiated CD4^+^ T cells (CD3+CD8-CD4+CD45RA+CCR7-CD27-). Data was acquired on a BD LSR II flow cytometer using the FACSDiva software (Becton Dickinson) and analysed using Flow Jo version 9 (Treestar).

### Cell sorting

Central, transitional and effector memory CD4^+^ T cells were sorted based on their expression of PD-1, TIGIT or LAG-3. The antibodies used for sorting were similar than those used for phenotyping with the exception of CD27-QDot655 (clone CLB-27/1, Invitrogen#Q10066). In a second set of experiments, total memory CD4^+^ T cells (CD3+CD4+CD45RA-) were sorted based on their expression of PD-1, TIGIT and LAG-3. Sorted cells were collected using an ARIA FACS sorter (Becton Dickinson).

### Isolation of total CD4^+^ T cells

Total CD4^+^ T cells were isolated from cryopreserved PBMCs using magnetic depletion as per the manufacturer’s protocol (Stem Cell Technologies, Vancouver, Canada).

### Quantification of integrated, total and 2-LTR circles HIV DNA, cell associated unspliced HIV RNA and Tat/rev inducible multiply spliced HIV RNA

Total CD4^+^ T cells or sorted CD4^+^ T cell subsets were used to measure the frequency of cells harboring HIV DNA (total, integrated and 2-LTR circles) by real time nested PCR as previously described [34] (S1A Text).

The CA-US RNA was measured by real time nested PCR as previously described [35]. The frequency of CD4^+^ T cells with inducible multiply spliced HIV RNA was determined using Tat/rev inducible limiting dilution assay (TILDA) [36].

### Statistical analysis

Data distributions were assessed through descriptive statistics and scatter plots. Negative binomial regression models were run for each set of comparisons with the percentage of CD4^+^ T cells expressing ICs being the predictor and the measure of HIV persistence the outcome. We chose this approach for reasons described previously [12, 35], and for consistency with those previous publications (S1B Text). The approach allowed us to fit models adjusting for the effects of absolute current or nadir CD4^+^ T-cell, which were examined for all combinations of IC predictors and HIV persistence outcome measures. In addition, the negative binomial regression models take into account that copies/input is measured with less precision when the number of copies is lower and when the amount of input is lower. The methods also permit proper quantitative use of instances where zero copies were present in the specimen assayed, without a need for ad hoc modifications to permit taking logarithms. We did not evaluate the results of alternative analysis methods and did not choose the methods post-hoc based on the results that they produced. Analyses were run in Stata version 13.1 (Stata Corp, College Station, TX).

For TILDA results analysis, we estimated the within-person fold difference in TILDA between the 2 cell subsets analyzed (memory CD4^+^ T cells expressing any versus none of the ICs) by fitting a maximum likelihood model to the raw data on numbers of positive and negative wells at each dilution (S1C Text).

## Results

### PD-1, TIGIT and LAG-3 are associated with markers of HIV persistence during ART

**Fig 1:**
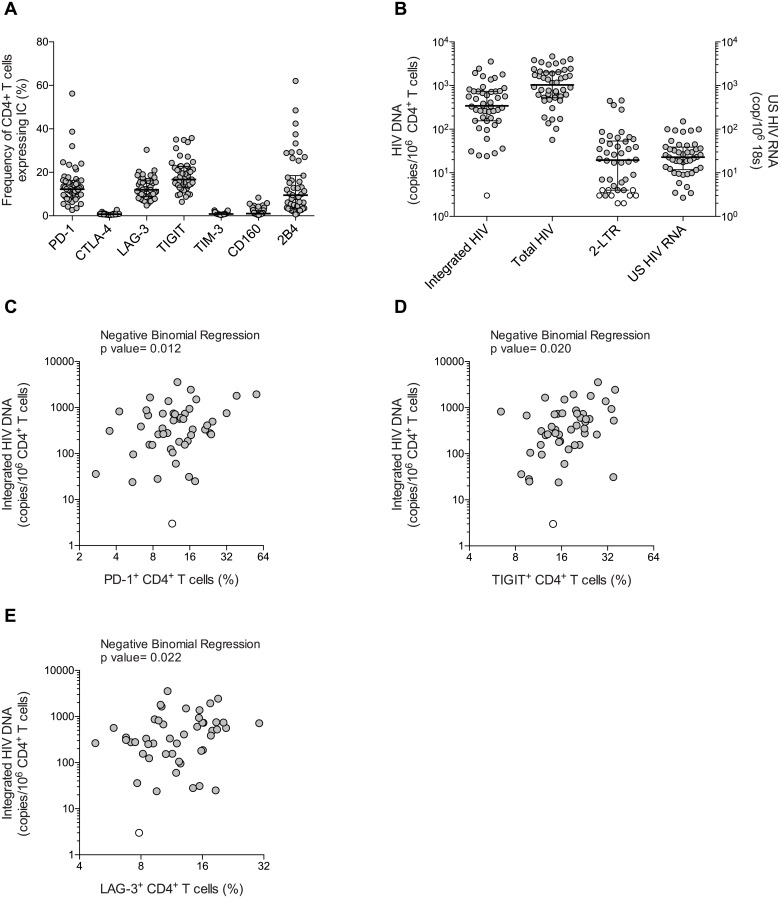
PD-1, TIGIT and LAG-3 are associated with virological markers of HIV persistence during ART. (A) Expression of 7 ICs on CD4^+^ T cells in individuals receiving suppressive ART (n = 48). Data is represented as percentage of CD4^+^ T cells and horizontal bars indicate median values with interquartile ranges. (B) Size of the HIV reservoir measured by integrated HIV DNA, total HIV DNA, 2-LTR circles represented as copies per million CD4^+^ T cells and cell-associated US HIV RNA represented as copies per million copies of 18S. Horizontal bars indicate median values with interquartile ranges and open circles represent the limit of detection in the negative samples (based on cell input). (C), (D), (E) Associations between the frequency of CD4^+^ T cells harboring integrated HIV DNA and the frequency of CD4^+^ T cells expressing PD-1, TIGIT and LAG-3, respectively. P values were obtained from negative binomial regression analysis. Effect sizes for the associations are as follows: (C) A 2-fold increase in the percentage of PD-1^+^ CD4^+^ T cell was associated with 1.43-fold increase in the frequency of CD4^+^ T cell harboring integrated HIV DNA, (D) a 2-fold higher percentage of TIGIT^+^ CD4^+^ T cell was associated with 1.91-fold higher integrated HIV DNA and (E) a 2-fold higher percentage of LAG-3^+^ CD4^+^ T cell was associated with 1.62-fold higher integrated HIV DNA. Open circles represent the limit of detection in the negative samples (based on cell input).

To determine the relationship between ICs and HIV persistence, 48 HIV-infected participants on suppressive ART for a median time (IQR) of 8.5 years (5.0–12.4) and a median CD4^+^ T-cell count (IQR) of 684 cells/μL (533–858) were recruited (Table 1). The expressions of 7 ICs on CD4^+^ T cells (PD-1, CTLA-4, LAG-3, TIGIT, TIM-3, CD160 and 2B4) were measured by multiparametric flow cytometry (S1 Fig). The frequencies of CD4^+^ T cells expressing these ICs were variable (median (IQR) of 16.7% (13.2–22.7), 12.2% (8.8–16.4), 12.0% (8.9–16.1), 9.5% (3.5–18.5), 1.1% (0.8–2.5), 0.8% (0.6–1.5) and 0.7% (0.6–1.0) for TIGIT, PD-1, LAG-3, 2B4, CD160, TIM-3 and CTLA-4 respectively) (Fig 1A).

**Table 1:** Clinical demographics of the cohorts.

Characteristics	Cohort 1 (N = 48)	Cohort 2 (N = 31)
**Gender,** n (%)		
**Male**	46 (96%)	29 (94%)
**Female**	1 (2%)	2 (6%)
**Transgender**	1 (2%)	0 (0%)
**Age, years** median (IQR)	57 (50–62)	50 (50–62)
**Viral load, cop/mL**	<50	<50
**Nadir CD4** ^**+**^ **T-cell count, cells/μl** median (IQR)	197 (110–285)^a^	42 (14–179)^b^
**Current CD4** ^**+**^ **T-cell count, cells/μl** median (IQR)	684 (530–862)	526 (420–702)
**Current CD8** ^**+**^ **T-cell count, cells/μl** median (IQR)	914 (639–1091)	830 (617–1135)
**CD4/CD8 ratio** median (IQR)	0.77 (0.57–1.17)	0.64 (0.40–0.91)
**ART, years** median (IQR)	8.5 (5.0–12.4)	8.8 (6.2–12.6)^c^

Data only available for (a) N = 45, (b) N = 27, (c) N = 24

The size of the HIV reservoir was determined by measuring the frequencies of CD4^+^ T cells harboring integrated HIV DNA, total HIV DNA and 2-LTR circles as well as cell-associated unspliced (CA-US) HIV RNA (Fig 1B and S1 Table). Total HIV DNA and cell-associated US HIV RNA were detected in all samples tested, whereas integrated HIV DNA and 2-LTR circles were detected in 98%, and 80% of the samples, respectively.

We evaluated the association between markers of HIV persistence and the frequencies of CD4^+^ T cells expressing ICs using a negative binomial regression model that was adjusted for current and nadir CD4^+^ T-cell counts when indicated (Table 2 and S2–S4 Tables). Using these tailored analytical methods for HIV reservoir measurements, we identified 3 ICs for which the expression on CD4^+^ T cells was statistically significantly associated with the frequency of CD4^+^ T cells harboring integrated HIV DNA, namely PD-1, TIGIT and LAG-3 (Fig 1C–1E and Table 2). These correlations persisted after adjusting for nadir CD4^+^ T-cell counts but were no longer significant after adjusting for current CD4^+^ T-cell count, a clinical parameter strongly associated with the size of the reservoir during ART [6,37,38].

**Table 2:** Negative binomial regression models to assess the relationship between integrated HIV DNA and IC expression on CD4^+^ T cells.

Outcome	Predictor^a^	Unadjusted		Adjusted for Current CD4		Adjusted for Nadir CD4	
		Result (95%CI)^c^ ^,^ ^d^	p-value^e^	Result (95%CI)	p-value	Result (95%CI)	p-value
Integrated HIV DNA ^b^	PD1^+^	1.43 (1.08 to 1.90)	**0.012**	1.17 (0.91 to 1.50)	0.220	1.39 (1.05 to 1.82)	**0.020**
	CTLA-4^+^	1.50 (0.90 to 2.51)	0.120	1.18 (0.73 to 1.91)	0.500	1.43 (0.88 to 2.35)	0.150
	LAG-3^+^	1.62 (1.07 to 2.45)	**0.022**	1.07 (0.69 to 1.65)	0.760	1.58 (1.07 to 2.35)	**0.023**
	TIGIT^+^	1.91 (1.11 to 3.28)	**0.020**	1.54 (0.91 to 2.60)	0.110	1.87 (1.10 to 3.16)	**0.020**
	TIM-3^+^	1.28 (0.83 to 1.97)	0.260	1.24 (0.85 to 1.80)	0.260	1.38 (0.89 to 2.14)	0.160
	CD160^+^	1.07 (0.82 to 1.39)	0.620	0.90 (0.69 to 1.17)	0.420	1.13 (0.86 to 1.48)	0.370
	2B4^+^	1.06 (0.83 to 1.36)	0.630	0.92 (0.71 to 1.18)	0.500	1.10 (0.87 to 1.39)	0.440

^a^ Percentage CD4^+^ T cells that express Immune Checkpoint Molecules

^b^ Integrated HIV DNA units (copies/million CD4^+^ T cells)

^c^ 95% CI = 95% confidence interval

^d^ Result interpretation: fold-change in the outcome (marker of HIV persistence) for each unit change of the predictor (Immune Checkpoint Molecules). All predictors were log2 transformed, so results here are interpreted as the change in the outcome for each doubling of the predictor

^e^ Statistically significant p values are <0.05 and are bold

The frequency of PD-1 expressing CD4^+^ T cells was also associated with the frequency of CD4^+^ T cells harboring total HIV DNA (S3 Table), but only marginally (1.23-fold effect, p = 0.07) when the model was adjusted for current CD4^+^ T-cell count. CA-US HIV RNA and 2-LTR circles did not show statistically significant correlation with any IC expression levels, with the exception of a negative association between the frequency of CD160^+^ CD4^+^ T cells and 2-LTR circles that remained statistically significant after adjusting for current and nadir CD4^+^ T-cell count (S3 and S4 Tables).

### Co-expression of PD-1, TIGIT and LAG-3 is a marker of HIV persistence during ART

**Fig 2:**
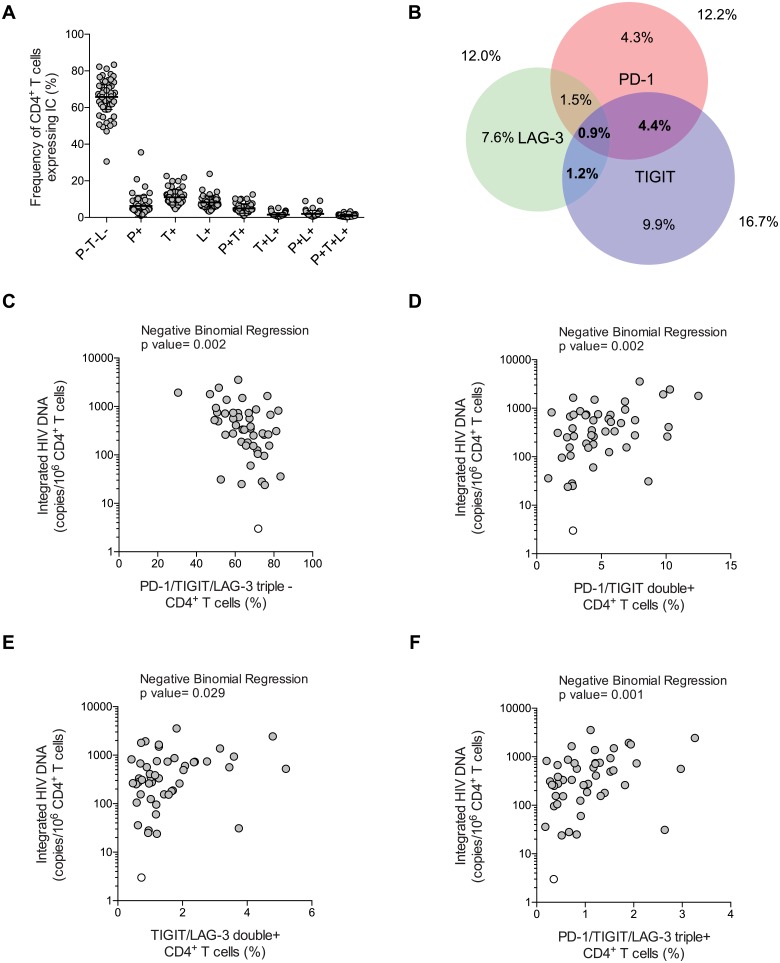
Co-expression of PD-1, TIGIT and LAG-3 is a marker of HIV persistence during ART. (A) Frequency of CD4^+^ T cells co-expressing PD-1 and/or TIGIT and/or LAG-3 (PD-1/TIGIT/LAG-3 triple–(P-T-L-), PD-1 single + (P+), TIGIT single + (T+), LAG-3 single + (L+), PD-1/TIGIT double + (P+T+), TIGIT/LAG-3 double + (T+L+), PD-1/LAG-3 double + (P+L+) and PD-1/TIGIT/LAG-3 triple + (P+T+L+)) determined by Boolean gating in cohort 1 (n = 48). Horizontal bars indicate median values with interquartile ranges. (B) Venn diagram showing the pattern of co-expression of PD-1, TIGIT and LAG-3. (C), (D), (E), (F) Associations between the frequency of CD4^+^ T cells harboring integrated HIV DNA and the frequency of CD4^+^ T cells expressing none of these markers (triple–), PD-1 and TIGIT (double +), TIGIT and LAG-3 (double +) and PD-1 and TIGIT and LAG-3 (triple +), respectively. P values were obtained from negative binomial regression analysis. Effect sizes for the associations are as follows: (C) 0.69-fold-change in integrated HIV DNA for 1 point increase in percentage of PD-1/TIGIT/LAG-3 triple—CD4^+^ T cells, (D) 1.18-fold-change in integrated HIV DNA for 1 point increase in percentage of PD-1/TIGIT double + CD4^+^ T cells, (E) 1.30-fold-change in integrated HIV DNA for 1 point increase in percentage of TIGIT/LAG-3 double + CD4^+^ T cells and (F) 1.94-fold-change in integrated HIV DNA for 1 point increase in percentage of PD-1/TIGIT/LAG-3 triple + CD4^+^ T cells. Open circles represent the limit of detection in the negative samples (based on cell input).

ICs are co-expressed on exhausted CD4^+^ and CD8^+^ T cells during untreated HIV infection [39]. Using a Boolean gating strategy, we determined the frequency of CD4^+^ T cells co-expressing PD-1 and/or TIGIT and/or LAG-3 in our cohort of 48 HIV-infected participants receiving suppressive ART (Fig 2A and 2B). The majority of CD4^+^ T cells did not express any of these markers (median (IQR) of 65.8% (59.0–72.4)) (S6 Table). Less than 10% (8.5%) of CD4^+^ T cells expressed more than one of these markers and 0.9% simultaneously expressed PD-1, TIGIT and LAG-3. We further assessed if the frequency of these discrete CD4^+^ T-cell subsets was associated with markers of HIV persistence. Using the negative binomial regression model, we found that the frequency of CD4^+^ T cells not expressing PD-1, TIGIT and LAG-3 was strongly and negatively correlated to the frequency of CD4^+^ T cells harboring integrated HIV DNA (p = 0.002, Table 3 and Fig 2C). Conversely, the frequency of CD4^+^ T cell co-expressing PD-1, TIGIT and LAG-3 was strongly and positively associated with the frequency of CD4^+^ T cells harboring integrated HIV DNA (p = 0.001, Table 3 and Fig 2F). Interestingly, the frequencies of CD4^+^ T cells co-expressing TIGIT with either PD-1 or LAG-3 were also positively associated with the frequency of CD4^+^ T cells harboring integrated HIV DNA (p = 0.002 and p = 0.029 respectively, Table 3 and Fig 2D and 2E). Although several of these associations were less or no longer statistically significant when the model was adjusted for the current CD4^+^ T-cell count, the association between the size of the HIV reservoir and the frequency of triple positive cells (PD-1+, LAG-3+ and TIGIT+) remained statistically significant after adjustment (p = 0.038). Adjusting for duration of ART did not produce any substantial changes to the results from the unadjusted analysis (all fold-effects adjusted for ART duration within 7% of those unadjusted). All together, these results indicate that the co-expression of PD-1, TIGIT and LAG-3 identifies a unique subset of CD4^+^ T cells that strongly predicts the frequency of cells harboring integrated HIV DNA during ART.

**Table 3:** Negative binomial regression models to assess the relationship between Integrated HIV DNA and the frequency of CD4^+^ T cells expressing PD-1 and/or TIGIT and/or LAG-3.

Outcome	Predictor^a^	Unadjusted		Adjusted for Current CD4		Adjusted for Nadir CD4		Adjusted for ART duration	
		Result (95%CI) ^c^ ^,^ ^d^	p-value^e^	Result (95%CI)	p-value	Result (95%CI)	p-value	Result (95%CI)	p-value
Integrated HIV DNA^b^	PD-1/TIGIT/LAG-3 triple –^f^	0.69 (0.54 to 0.87)	**0.002**	0.82 (0.65 to 1.03)	0.084	0.70 (0.56 to 0.86)	**0.001**	0.68 (0.54–0.85)	**0.001**
	PD-1 single +^f^	1.24 (0.90 to 1.70)	0.187	1.09 (0.80 to 1.48)	0.578	1.28 (0.94 to 1.75)	0.118	1.32 (0.93–1.88)	0.12
	TIGIT single +	1.06 (0.99 to 1.14)	0.106	1.05 (0.98 to 1.12)	0.210	1.06 (0.99 to 1.13)	0.111	1.06 (0.98–1.14)	0.13
	LAG-3 single +	1.00 (0.95 to 1.05)	0.963	0.97 (0.92 to 1.02)	0.176	1.00 (0.94 to 1.05)	0.848	1.00 (0.95–1.05)	0.86
	PD-1/TIGIT double +	1.18 (1.07 to 1.31)	**0.002**	1.13 (1.00 to 1.26)	**0.042**	1.17 (1.06 to 1.30)	**0.003**	1.19 (1.07–1.32)	**0.001**
	TIGIT/LAG-3 double +	1.30 (1.03 to 1.64)	**0.029**	1.16 (0.97 to 1.38)	0.100	1.27 (1.04 to 1.55)	**0.018**	1.29 (1.02–1.65)	**0.037**
	LAG-3/PD-1 double +	1.08 (0.96 to 1.23)	0.206	1.01 (0.88 to 1.16)	0.910	1.09 (0.96 to 1.25)	0.175	1.11 (0.96–1.23)	0.15
	PD-1/TIGIT/LAG-3 triple +	1.94 (1.33 to 2.83)	**0.001**	1.45 (1.02 to 2.05)	**0.038**	1.86 (1.26 to 2.72)	**0.002**	1.92 (1.31–2.81)	**0.001**

^a^ Percentage CD4^+^ T cells that express Immune Checkpoint Molecules

^b^ Integrated HIV DNA units (copies/million CD4^+^ T cells)

^c^ 95% CI = 95% confidence interval

^d^ Result interpretation: fold-change in the outcome (marker of HIV persistence) for each one point increase in the percent of cells expressing the predictor (PD-1 and/or TIGIT and/or LAG-3)

^e^ Statistically significant p values are <0.05 and are bold

^f^ These predictors vary more than others, so results are scaled to be per 10 point increase in percent of cells, rather than per one point increase

When the negative binomial regression model was used to assess the relationship between Total HIV DNA, 2-LTR circles, CA-US RNA and the frequency of CD4^+^ T cells expressing PD-1 and/or TIGIT and/or LAG-3, no association show statistically significant, with the exception of the frequency of CD4^+^ T cells harboring total HIV DNA and the frequency of PD-1 single + (p = 0.005, 1.10-fold-change in total HIV DNA for 1 point increase in percentage of PD-1 single + CD4^+^ T cells) and PD-1/TIGIT double + CD4+ T cells (p = 0.017, 1.40-fold-change in total HIV DNA for 1 point increase in percentage of PD-1/TIGIT double + CD4^+^ T cells).

### Expressions of PD-1, TIGIT and LAG-3 are associated with HIV-infected cells in distinct memory CD4^+^ T-cell subsets during ART

**Fig 3:**
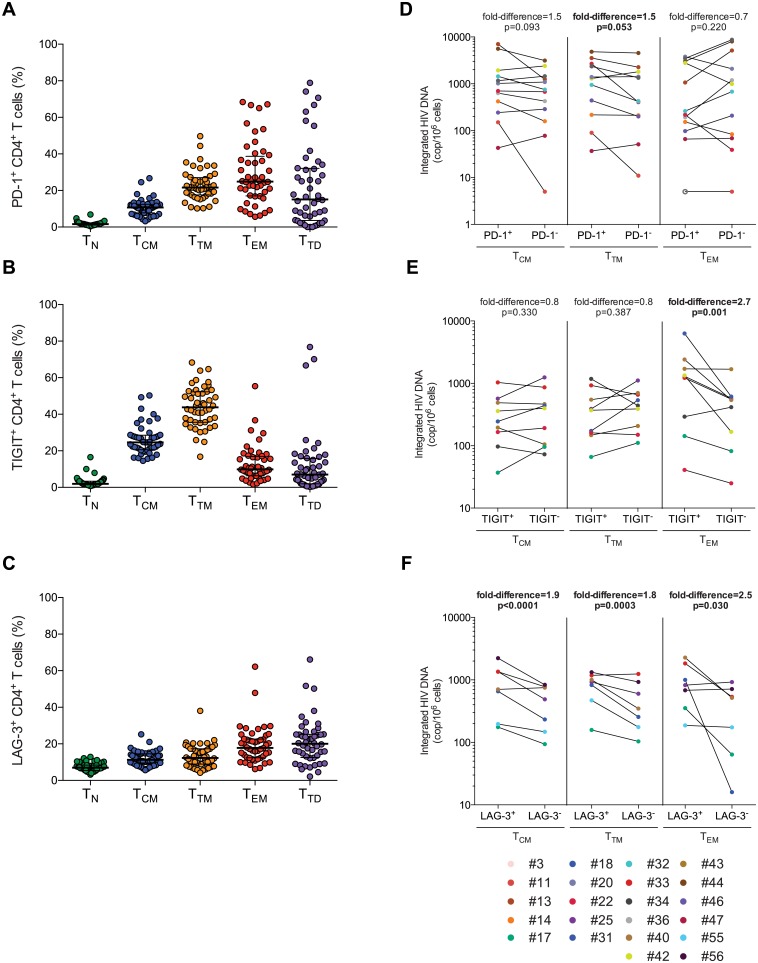
PD-1, TIGIT and LAG-3 identify HIV-infected cells in distinct memory CD4^+^ T-cell subsets during ART. (A), (B), (C) Frequencies of memory CD4^+^ T-cell subsets (naïve (T_N_), central memory (T_CM_), transitional memory (T_TM_), effector memory (T_EM_) and terminally differentiated (T_D_)) expressing PD-1, TIGIT or LAG-3, respectively. Horizontal bars indicate median values with interquartile ranges. (D), (E), (F) Frequencies of cells harboring integrated HIV DNA in T_CM_, T_TM_ and T_EM_ CD4^+^ T-cell subsets sorted based on their expression of PD-1 (n = 12), TIGIT (n = 9) or LAG-3 (n = 7), respectively. Results are expressed as the HIV copy number in million cells of a given subset. P values were obtained from negative binomial regression analysis. Significant differences (p<0.05) are designated by a p value in bold. Open circles represent the limit of detection in the negative samples (based on cell input).

HIV persists preferentially in memory CD4^+^ T-cell subsets [6–8]. To determine the role played by ICs in each individual CD4^+^ T-cell memory subset, we first analyzed the expression of PD-1, TIGIT and LAG-3, the 3 ICs we identified to be associated with HIV persistence, on naïve (T_N_), central memory (T_CM_), transitional memory (T_TM_), effector memory (T_EM_) and terminally differentiated (T_TD_) cells in 48 HIV-infected participants (Cohort 1: clinical characteristics in Table 1) (Fig 3A, 3B and 3C respectively). As expected, T_N_ cells expressed low levels of these ICs. The frequency of CD4^+^ T cells expressing PD-1 or LAG-3 increased with differentiation, with T_EM_ cells displaying the highest levels of expression of these markers. The highest frequency of TIGIT^+^ cells was found within the T_TM_ subset. These results demonstrated that the subsets of memory cells that were previously shown to harbor persistent HIV during ART express PD-1, TIGIT and LAG-3.

To determine whether PD-1, TIGIT and LAG-3 identify cells more likely to carry persistent HIV in virally suppressed participants, individual memory CD4^+^ T-cell subsets were sorted based on their expression of PD-1, TIGIT or LAG-3 in a subset of subjects who underwent leukapheresis (Cohort 2: clinical characteristics in Table 1) and the results were analyzed by negative binomial regression model (S5 Table). The frequency of cells harboring integrated HIV DNA was moderately higher in PD-1 expressing T_TM_ when compared to their PD-1 negative counterparts (p = 0.053, fold-difference = 1.5) (Fig 3D). T_EM_ cells expressing TIGIT were enriched for integrated genomes when compared to their TIGIT^-^ counterparts (p = 0.001, fold-difference = 2.7) (Fig 3E). Finally, all the memory CD4^+^ T-cell subsets (T_CM_, T_TM_ and T_EM_ cells) expressing LAG-3 were enriched for integrated HIV DNA when compared to their negative counterparts (p<0.0001, fold-difference = 1.9, p = 0.003, fold-difference = 1.8 and p = 0.030, fold-difference = 2.5 respectively) (Fig 3F). All together these results indicate that PD-1, TIGIT and LAG-3 enrich for infected cells in distinct memory CD4^+^ T-cell subsets in individuals on ART. We calculated the contribution of cells expressing PD-1, TIGIT or LAG-3 to the total reservoir by taking into account the frequency of these subsets within the CD4 compartment and their relative infection frequencies. The mean contributions of CD4^+^ T cells expressing PD-1, TIGIT and LAG-3 were 29%, 34% and 31%, respectively (S2 Fig). As a comparator, T_CM_, T_TM_ and T_EM_ cells contributed 43%, 27% and 24% to the pool of infected cells in these same virally suppressed individuals. These data indicate that a third of the reservoir is encompassed in cells expressing each individual marker.

### Co-expression of PD-1, TIGIT and LAG-3 highly enriches in HIV-infected memory CD4^+^ T cells during ART

**Fig 4:**
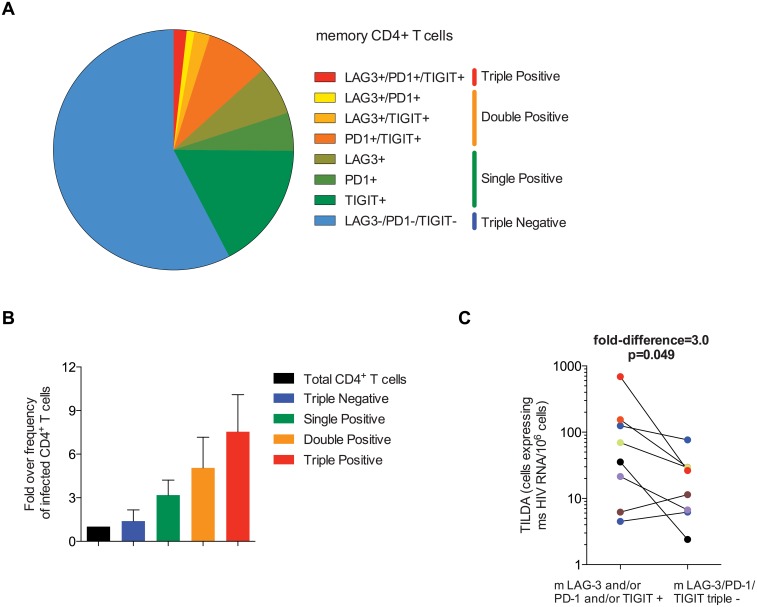
Co-expression of PD-1, TIGIT and LAG-3 identifies HIV-infected cells during ART. (A) Pie chart representing the frequencies of memory CD4^+^ T cells co-expressing PD-1 and/or TIGIT and/or LAG-3 in cohort 1 (n = 48). Coloured bars on the right side designate categories of ICs expressing cells: Triple -: PD-1/TIGIT/LAG-3 triple—in blue; single +: PD-1 single +, TIGIT single +, LAG-3 single + in green; double +: PD-1/TIGIT double +, TIGIT/LAG-3 double +, PD-1/LAG-3 double + in orange and triple +: PD-1/TIGIT/LAG-3 triple + in red. (B) Frequency of memory CD4^+^ T cells harboring integrated HIV DNA represented as a fold change over frequency in total CD4^+^ T cells. Mean values and standard deviations from 5 independent donors are represented (n = 5). (C) Frequency of cells harboring inducible msRNA measured by TILDA in memory CD4^+^ T cells expressing any (i.e. at least one) versus none of PD-1, TIGIT or LAG-3 (mLPT+ and mLPT- respectively). P value and fold-difference were obtained from a maximum likelihood model.

We then determined if the combination of PD-1, TIGIT and LAG-3 would further enrich memory CD4^+^ T cells for HIV-infected cells during ART. The average frequency of cells expressing 0, 1, 2 or 3 of these markers in the memory CD4^+^ T compartment (CD45RA-) from our cohort of 48 individuals (Table 1) indicated that an average of 33% of memory CD4^+^ T cells expressed one of the 3 IC only, 12% expressed 2 and 2% expressed the 3 markers simultaneously. Large numbers of memory CD4^+^ T cells were sorted based on their expression of PD-1, TIGIT and LAG-3 from 5 individuals. The combination of these 3 markers allowed us to sort eight subsets of cells to high purity, namely PD-1/TIGIT/LAG-3 triple -, PD-1 single +, TIGIT single +, LAG-3 single +, PD-1/TIGIT double +, TIGIT/LAG-3 double +, PD-1/LAG-3 double + and PD-1/TIGIT/LAG-3 triple + cells. The frequency of cells harboring integrated HIV DNA was measured by qPCR in each sorted subset (S3 Fig) and the mean frequency for each category was calculated relative to total CD4^+^ T cells (Fig 4B). Memory CD4^+^ T cells showed a gradual enrichment in HIV-infected cells when expressing an increasing number of ICs. Memory CD4^+^ T cells expressing simultaneously PD-1, TIGIT and LAG-3 were enriched for HIV-infected cells up to 10 times more when compared to total CD4^+^ T cells, with a median fold increase (IQR) of 8.15 (4.92–9.59). These results demonstrated that memory CD4^+^ T cells expressing a combination of ICs were highly enriched for integrated HIV DNA during ART.

As the majority of HIV genomes, even when integrated, are defective [40], we assessed if PD-1, TIGIT and LAG-3 identify cells in which HIV production can be induced. As the frequency of triple positive cells was too low to perform this experiment, we sorted memory CD4^+^ T cells (CD45RA-) expressing any (i.e. at least one) versus none of PD-1, TIGIT or LAG-3 (mLPT+ and mLPT- respectively). We measured the frequency of cells in each population that transcribe multiply spliced HIV RNA molecules upon induction with PMA/ionomycin using the Tat/rev induced limiting dilution assay (TILDA) [36]. Tat/rev transcripts were detectable by TILDA in both cell subsets from all of the 8 individuals tested. The rate of inducible virus per million cells was estimated in our maximum likelihood model to average 3.0-fold higher in mLPT+ cells than in mLPT- cells from the same participant (95% CI 1.0 to 9.0, p = 0.049, S1C Text) (Fig 4C). Taking into account the frequency of these cell subsets, the contribution of cells expressing at least one of these markers to the total pool of memory CD4^+^ T cells infected with inducible HIV genomes was calculated. This contribution ranged from 30 to 98% (median of 76%), indicating that the majority of inducible HIV genomes were found in memory CD4^+^ T cells expressing at least one of these markers. These experiments provide evidence that memory CD4^+^ T cells expressing PD-1, TIGIT and/or LAG-3 are enriched for HIV-infected CD4^+^ T cells harboring inducible proviruses during ART.

## Discussion

In this study, we identified PD-1, TIGIT and LAG-3 as novel markers of cells that are more frequently infected in HIV-infected individuals receiving suppressive ART. Co-expression of the 3 ICs identified a unique subset of CD4^+^ T cells that was strongly associated with the size of the HIV reservoir and that was highly enriched for integrated HIV DNA. Finally, our results provide evidence that memory CD4^+^ T cells expressing at least one of these markers are the major contributors to the pool of inducible HIV genomes during ART.

The frequencies of CD4^+^ T cells expressing PD-1, CTLA-4, LAG-3 and TIM-3 were similar to those reported by other groups [41–44], indicating that the cohort of participants used for this study is likely to be representative of the HIV population receiving suppressive ART. In addition, the association between PD-1 and TIGIT expression on CD4^+^ T cells and the frequency of CD4^+^ T cells carrying HIV proviruses was in agreement with previously reported findings [6,24,41].

We found positive associations between the expression of PD-1, LAG-3 and TIGIT in CD4^+^ T cells and the frequency of cells harboring integrated HIV DNA. Of note, these three markers showed the strongest inverse associations with CD4^+^ T cell counts among the 7 markers we examined, suggesting a link between T cell homeostasis and HIV persistence (S4A, S4B and S4C Fig). The associations between individual IC expression and HIV persistence marker were substantially smaller and no longer statistically significant after adjusting for current CD4^+^ T-cell count. These findings from the negative binomial regression models suggest that the current CD4^+^ T-cell count is an important predictor of the size of the HIV reservoir when measured as the frequency of cells harboring proviral genomes [6,37]. Importantly, and in contrast to cells expressing a single marker, the frequency of cells co-expressing simultaneously PD-1, TIGIT and LAG-3 was strongly associated with the size of the reservoir and remained after adjusting for nadir and current CD4^+^ T-cell counts. This result reinforces the possibility of a direct—and maybe synergistic—role for these molecules in HIV persistence during ART. In addition, the frequency of CD4^+^ T cells co-expressing simultaneously PD-1, TIGIT and LAG-3 positively correlated with the frequency of CD4^+^ T cells expressing HLADR/CD38 (p = 0.003, r = 0.42) and Ki67 (p = 0.022, r = 0.33) (S1D and S1E Text and S4D and S4E Fig). These associations suggest that the persistence of the small pool of cells expressing the 3 markers is associated with T cell activation and proliferation.

Interestingly, we observed a strong negative association between CD160 expression and 2-LTR circles. Notably, this correlation remained after adjusting for current and nadir CD4^+^ T cell counts. A possible explanation for these findings is that CD160^+^ cells may be preferential targets for infection and depletion during ART, which would explain the strong negative association between the frequency of CD160^+^ CD4^+^ T cells and a putative marker of persistent viral replication.

By sorting T_CM_, T_TM_ and T_EM_ cells expressing PD-1, TIGIT and LAG-3, we observed that cells expressing these markers were enriched for HIV-infected cells in different memory CD4^+^ T-cells subsets during ART. While LAG-3 enriched for integrated HIV DNA in all memory subsets (T_CM_, T_TM_, and T_EM_), PD-1 and TIGIT enriched for HIV genomes exclusively in T_TM_ and T_EM_ cells, respectively. These observations suggest that ICs may exert different pro-latency effects in subsets endowed with distinct proliferative and activation status. One may hypothesize that different ICs provide infected cells with different selective advantage to persist by counteracting distinct stimuli specific to an individual memory cell subset. Further investigations will be needed to characterize the mechanisms by which these ICs may specifically contribute to HIV persistence within these distinct subsets.

Overall, the majority of inducible HIV genomes were found in memory CD4^+^ T cells expressing at least one of these markers (median of 76%). Although triple negative cells also contain inducible HIV genomes, our data provide evidence that there is an enrichment for inducible viral genomes in CD4^+^ T cells expressing these markers.

Importantly, we found a gradual enrichment in integrated HIV DNA in cells that express multiple ICs simultaneously. This observation mirrors the synergistic mechanisms of action of these receptors to dampen T cell functions. Indeed, LAG-3 and PD-1 are commonly co-expressed on exhausted or dysfunctional T cells in models of chronic infections [45], autoimmune diseases [46], and cancers [47,48]. Potential synergistic functions were highlighted in murine models of autoimmune diseases [49]. Anti-LAG-3 blocking mAb has recently entered clinical testing in cancer in monotherapy or in combination therapy with anti-PD-1 (NCT01968109). Additionally, TIGIT is co-expressed with PD-1 on activated CD8^+^ tumor-infiltrating lymphocytes from patients with melanoma [50]. Blockages of TIGIT and PD-1 synergize to improve T cell proliferation, cytokines production and degranulation *in vivo* in melanoma treatment and *in vitro* in HIV infection [24,50]. All together, these studies indicate that ICs can synergize to repress T cell functions and suggest that these synergies may also play a role in HIV persistence during ART.

The expression of PD-1, LAG-3 and TIM-3 on CD4^+^ and CD8^+^ T cells prior to ART was recently identified as a strong predictor of time to viral rebound after treatment interruption in the SPARTAC study [43]. It is possible that CD4^+^ T cells expressing these markers before ART represent a preferential niche for the establishment of a stable reservoir for HIV and that latently infected cells expressing these markers preferentially persist during ART, as suggested by our observations. In our study, we identified a discrete subset of CD4^+^ T cells co-expressing PD-1, TIGIT and LAG-3 as an important predictor of the frequency of cells harboring integrated HIV DNA during ART. Of note, the expression of TIGIT before ART initiation was not measured in the SPARTAC study and further studies will be needed to determine if this IC could also represent a pre-ART predictor of viral rebound.

Our data provide a rationale for the use of immune checkpoint blockers (ICBs) to target latently infected cells during ART. Targeting ICs by ICBs, a novel class of molecules in development in oncology, may have a double benefit in the context of HIV remission by both targeting latently infected cells and restoring HIV-specific T cell immunity. By enhancing T cell activation and increasing viral transcription, ICBs may facilitate HIV reactivation in latently infected cells when used alone or in combination with latency reversing agents. The anti-CTLA-4 antibody iplimumab was recently shown to significantly increase CA-US HIV RNA in an HIV-infected individual on ART, consistent with latency reversal [51]. An alternative mechanism of action of some ICBs would be to directly deplete cells expressing these markers, as observed with the anti-CTLA-4 ipilumimab, which induces direct elimination of CTLA-4^+^ regulatory T cells in tumor tissue in patients with melanoma [52]. Our results suggest that the administration of antibodies with effector functions targeting PD-1, LAG-3 and TIGIT may significantly reduce the size of the latent HIV reservoir during ART by targeting cells in which HIV persists.

Several limitations are associated with our study. We have not adjusted p-values for multiple comparisons, because such adjustment would neglect the biological relationships among our positive results and would require that each analysis detract from the others, rather than reinforcing one another when there is biological coherence [53] (S1F Text). Nevertheless, our evidence may be weaker than if it had arisen from a narrower set of analyses, and, in any case, additional studies will be needed to confirm the hypotheses supported by our results. Most of our analyses were performed using integrated HIV DNA as a marker of HIV persistence. We chose this readout as it was applicable to small subsets of CD4^+^ T cells on which measures of replication competent HIV cannot be performed. The majority of viral genomes persisting during ART are known to be defective [40,54], and although our experiments indicate that cells that express ICs can produce multiply spliced RNA upon activation (TILDA), they do not demonstrate that replication competent virus persists in these cells. In addition, our results are limited to circulating T cells. It is possible and indeed likely that the biology of ICs expression and HIV persistence will differ in tissues, particularly in secondary lymphoid tissues where many of the ligands for these receptors are likely to be expressed.

A better understanding of the nature of the cells that encompass the latent HIV reservoir is a prerequisite to the development of novel curative strategies. Despite similarities in their mechanisms of action, PD-1, TIGIT and LAG-3 are likely to be non-redundant in their functions. Blocking these pathways simultaneously may show synergies in latency reversal, as suggested by their synergistic activities in the restoration of T cell immunity.

## Supporting Information

S1 Text Materials & Methods supporting information. (DOCX)

S1 Fig Gating strategy of 7 ICs on CD4^+^ T cells. Expression of PD-1, CTLA-4, LAG-3, TIGIT, TIM-3, CD160 and 2B4 on CD4^+^ T cells in individuals receiving suppressive ART (n = 48). Representative dot plots from one participant.
(EPS)

S2 Fig Contribution of ICs expressing cells to the HIV reservoir pool. The contribution of cells expressing PD-1 (A), TIGIT (B) or LAG-3 (C) to the total HIV reservoir by taking into account the frequency of these subsets within the CD4 compartment and their relative infection frequencies (n = 12, n = 9 and n = 7, respectively). Horizontal bars indicate median values.
(EPS)

S3 Fig Frequency of memory CD4^+^ T cells harboring integrated HIV DNA in PD-1/TIGIT/LAG-3 triple–(LPT-), PD-1 single + (P), TIGIT single + (T), LAG-3 single + (L), PD-1/TIGIT double + (PT), TIGIT/LAG-3 double + (LT), PD-1/LAG-3 double + (LP) and PD-1/TIGIT/LAG-3 triple + (LPT+) cells. Raw data from the 5 subjects presented in Fig 4B.
(EPS)

S4 Fig PD-1, TIGIT and LAG-3 are associated with markers of activation/proliferation. (A), (B), (C) Associations between the current CD4^+^ T cell counts and the frequency of CD4^+^ T cells expressing PD-1, TIGIT and LAG-3, respectively. P, r values were obtained from Spearman’s ranked analysis. (D), (E) Associations between the frequency of CD4^+^ T cells co-expressing PD-1, TIGIT and LAG-3 and the frequencies of CD4^+^ T cells expression HLA-DR/CD38 and Ki67 respectively. P, r values were obtained from Spearman’s ranked analysis.
(EPS)

S1 Table Virological markers of HIV persistence. (DOCX)

S2 Table Negative binomial regression models to assess the relationship between Total HIV DNA and Immune Checkpoints expression on CD4^+^ T cells. (DOCX)

S3 Table Negative binomial regression models to assess the relationship between 2-LTR circles and Immune Checkpoints expression on CD4^+^ T cells. (DOCX)

S4 Table Negative binomial regression models to assess the relationship between cell-associated US HIV RNA and Immune Checkpoints expression on CD4^+^ T cells. (DOCX)

S5 Table Negative binomial regression models to compare integrated HIV DNA in cells expressing the Immune Checkpoint Molecule with integrated HIV DNA in cells not expressing the Immune Checkpoint Molecule. (DOCX)

S6 Table Frequencies of ICs on CD4^+^ T cells in cohort 1 (n = 48). (DOCX)
