# Supplementary material for: CD4+ T Cells Expressing PD-1, TIGIT and LAG-3 Contribute to HIV Persistence during ART
Source: PLoS Pathog. 2016 Jul 14;12(7):e1005761. doi: 10.1371/journal.ppat.1005761 (PMC4944956; doi:10.1371/journal.ppat.1005761)
Supplement: S1 Text — (DOCX) [file ppat.1005761.s001.docx]

**S1 Text**

**Materials & Methods A: Quantification of integrated, total and 2-LTR circles HIV DNA**

Total CD4^+^ T cells or sorted CD4^+^ T cell subsets were used to measure the frequency of cells harboring HIV DNA (total, integrated and 2-LTR circles) by real time nested PCR. Briefly, cell pellets were resuspended in a lysis buffer (10mM Tris-HCl pH 8.0, 50nM KCl, 400 µg/mL Proteinase K, Invitrogen) at the appropriate concentrations (5-10x10^6^ cells/mL) and digested for 12-16 hours at 55°C in a heating shaker. Proteinase K was inactivated by heating the digested samples at 95°C for 5 minutes. 15 µl of the cell lysate were used in all pre-amplification reactions. In all PCR reactions, primers specific for the human CD3 gene (HCD3OUT5’ and HCD3OUT3’) were used to quantify the exact number of cells present in the reaction tube. Pre-amplification of total HIV DNA and the CD3 gene were carried out in a 50 µL reaction mixture comprising 1X Taq polymerase Buffer (Invitrogen), 3 mM MgCl_2_, 300 µM dNTP (Invitrogen), 300 nM of each of the 4 primers (ULF1, UR1, HCD3OUT5’ and HCD3OUT3’) and 2.5U Taq polymerase (Invitrogen). The first round PCR cycle conditions were as follows: a denaturation step of 8 min at 95°C, 12 cycles of amplification (95°C for 1min, 55°C for 40 sec, 72°C for 1 min), followed by a final elongation step at 72°C for 15 min.

Integrated HIV genomes were amplified by using the same mix as total HIV DNA with the exception of the reverse primer (UR1), which was replaced by the Alu 1 and Alu 2 Primers (300 nM each) and the concentration of ULF1 that was reduced (150 nM). Given the high number of Alu elements within the human genome, abundant amplifications of inter-Alu sequences occurred simultaneously with the amplification of Alu-LTR sequences. To remain in the exponential phase, only 12 cycles of amplification were performed. The PCR cycle conditions were as follows: denaturation step of 8 min at 95°C, 12 cycles of amplification (95°C for 1 min, 55°C for 1 min, 72°C for 10 min), followed by an elongation step of 15 min at 72°C.

2-LTR circles sequences were amplified with a mix containing the CD3 primers together with the ULLTRF1 and ULTRR1 primers (all at 300 nM). The PCR conditions were as follows: denaturation step of 8 min at 95°C, 16 cycles of amplification (95°C for 30 sec, 55°C for 30 sec, 72°C for 1 min), followed by an elongation step of 15 min at 72°C. All pre-amplifications were carried out on a Mastercycler pro-S instrument (Eppendorf).

The second rounds of PCR were carried out in real-time on the RotorGene Q instrument (Qiagen) with the Rotor Gene Probe Master Mix (Qiagen) following the manufacturer’s instructions. All reactions (Total HIV DNA, Integrated HIV DNA, 2-LTR circles and CD3) were performed in a final volume of 20 µL containing 6.4 µL of a 1/10 dilution of the first PCR products. 1250 nM of the appropriate sets of primers (Lambda T and UR2 for total and integrated HIV, Lambda T and ULTRR2 for 2-LTR circles, and HCD3IN5’ and HCD3IN5’ for CD3) were added to the Rotor Gene Probe Master Mix. 200 nM of the UHIV Taqman probe was added to the total and integrated HIV DNA reactions, whereas the same concentration of the U2LTR Taqman probe was used for the 2-LTR reaction. For CD3 quantification, 200 nM of the CD3 Taqman probe was used. The same amplification steps were used for all reactions: a denaturation step (95°C for 4 min), followed by 40 cycles of amplification (95°C for 3 sec, 60°C for 10 sec).

**Materials & Methods B: Negative binomial regression model**

The negative binomial regression models allowed us to fit models adjusting for the effects of absolute current or nadir CD4^+^ T-cell, which were examined for all IC predictors of all HIV persistence outcome measures and treatment duration for the co-expression of ICs (Table 3). In addition, the negative binomial regression models take into account that copies/input is measured with less precision when the number of copies is lower and when the amount of input is lower. The methods also permit proper quantitative use of instances where zero copies were present in the specimen assayed, without a need for ad hoc modifications to permit taking logarithms. The percentage of CD4+ T cells expressing ICs were log transformed in all models.

**Materials & Methods C: TILDA statistical analysis**

The approach used for TILDA results analysis accounts for greater relative uncertainty for lower rates of inducible virus and for assays done with fewer replicates. The model included a random person effect and a parameter for the logarithm of the fold difference of mLPT+ (memory CD4+ T cells expressing PD-1, TIGIT or LAG-3) versus mLPT- (memory CD4+ T cells not expressing PD-1, TIGIT and LAG-3) in rates of inducible virus. Addition of a random effect to allow the mLPT+ versus mLPT- difference to vary from person to person improved the fit to the data, so we report results from that model. We confirmed the confidence interval and p-value by obtaining similar values by profile likelihood and likelihood ratio testing. In response to a reviewer’s request, we performed TILDA on sorted cells from two additional participants, increasing the sample size from 6 to 8. These results should be interpreted with caution, because increasing sample size in response to initial results can increase type I error.

**Materials & Methods D: Measure of the expression of activation/proliferation markers**

The same antibody backbone as for ICs panels was used to measure activation/proliferation markers in subsets of memory CD4+ T cells. CD38-PE (clone HIT2, BD#555460), HLA-DR-PerCP (clone L243, BD#347364), CD127-PE-CF594 (clone hIL-7R-M21, BD#562397) and intracellular Ki67-FITC (BD#556026) were added to this antibody backbone.

**Materials & Methods E: Activation/proliferation markers statistical analysis**

Spreaman’s ranked test was used to determine correlations between ICs expression on CD4 T cells and activation/proliferation markers.

**Materials & Methods F: Adjustment for multiple comparisons**

Although we examine many differences and associations, we report nominal p-values, without adjustment for multiple testing. Standard methods of such adjustment would be focused on avoidance of one or more results with p<0.05 in the case where all differences are truly zero (1-3), which is an unrealistic hypothesis about the state of nature in our situation. In addition, adjustment would require that each result detract from the others, but there are clear biological relationships among many of the associations that we examine, and these permit coherent sets of findings to reinforce each other rather than detract from one another. Thus, we believe that multiple comparison adjustment would not be appropriate for many of the results in this paper (4).

**Reference**

1. Rothman KJ. No adjustments are needed for multiple comparisons. Epidemiology. 1990 Jan;1(1):43–6.

2. Savitz DA, Olshan AF. Multiple comparisons and related issues in the interpretation of epidemiologic data. Am J Epidemiol. 1995 Nov 1;142(9):904–8.

3. Perneger TV. What's wrong with Bonferroni adjustments. BMJ. 1998 Apr 18;316(7139):1236–8.

4. Bacchetti P. Peer review of statistics in medical research: the other problem. BMJ. 2002 May 25;324(7348):1271–3.
