# Supplementary material for: CD4+ T Cells Expressing PD-1, TIGIT and LAG-3 Contribute to HIV Persistence during ART
Source: PLoS Pathog. 2016 Jul 14;12(7):e1005761. doi: 10.1371/journal.ppat.1005761 (PMC4944956; doi:10.1371/journal.ppat.1005761)
Supplement: S1 Table — (DOCX) [file ppat.1005761.s006.docx]

**S1 Table:** Virological markers of HIV persistence

| Virological marker | Frequency of non-zero measurements | Median of non-zero measurements | IQR of non-zero measurements |
| --- | --- | --- | --- |
| Integrated HIV DNA | 98% | 350 | 156-739 |
| Total HIV DNA | 100% | 1039 | 526-2055 |
| 2-LTR circles | 80% | 29 | 9-64 |
| US HIV RNA | 100% | 23 | 12-39 |
