# Supplementary material for: CD4+ T Cells Expressing PD-1, TIGIT and LAG-3 Contribute to HIV Persistence during ART
Source: PLoS Pathog. 2016 Jul 14;12(7):e1005761. doi: 10.1371/journal.ppat.1005761 (PMC4944956; doi:10.1371/journal.ppat.1005761)
Supplement: S2 Table — (DOCX) [file ppat.1005761.s007.docx]

**S2 Table:** Negative binomial regression models to assess the relationship between Total HIV DNA and Immune Checkpoints expression on CD4^+^ T cells.

| Outcome | Predictor^a^ | Unadjusted | | Adjusted for Current CD4 | | Adjusted for Nadir CD4 | |
| --- | --- | --- | --- | --- | --- | --- | --- |
|  |  | **Result**  **(95%CI) ^c, d^** | **p-value^e^** | **Result**  **(95%CI)** | **p-value** | **Result**  **(95%CI)** | **p-value** |
| Total HIV DNA^b^ | PD1^+^ | 1.30 (1.04 to 1.62) | **0.021** | 1.23 (0.98 to 1.55) | 0.072 | 1.28 (1.02 to 1.59) | **0.031** |
|  | CTLA-4^+^ | 0.82 (0.46 to 1.49) | 0.520 | 0.78 (0.45 to 1.36) | 0.380 | 0.82 (0.46 to 1.46) | 0.500 |
|  | LAG-3^+^ | 0.89 (0.62 to 1.28) | 0.530 | 0.75 (0.50 to 1.11) | 0.150 | 0.85 (0.57 to 1.26) | 0.410 |
|  | TIGIT^+^ | 1.29 (0.82 to 2.02) | 0.270 | 1.19 (0.76 to 1.85) | 0.440 | 1.25 (0.79 to 1.96) | 0.340 |
|  | TIM-3^+^ | 1.25 (0.91 to 1.73) | 0.170 | 1.26 (0.92 to 1.72) | 0.150 | 1.35 (0.99 to 1.85) | 0.058 |
|  | CD160^+^ | 1.01 (0.82 to 1.24) | 0.960 | 0.94 (0.75 to 1.18) | 0.570 | 1.03 (0.83 to 1.26) | 0.820 |
|  | 2B4^+^ | 1.03 (0.87 to 1.23) | 0.730 | 0.98 (0.80 to 1.19) | 0.810 | 1.05 (0.88 to 1.25) | 0.610 |

^a^ Percentage CD4^+^ T cells that express Immune Checkpoint Molecules

^b^ Total HIV DNA units (copies/million CD4^+^ T cells)

^c^ 95% CI = 95% confidence interval

^d^ Result interpretation: fold-change in the outcome (marker of HIV persistence) for each two-fold increase in the predictor (Immune Checkpoint Molecules)

^e^ Statistically significant p values are <0.05 and are bold
