# Supplementary material for: CD4+ T Cells Expressing PD-1, TIGIT and LAG-3 Contribute to HIV Persistence during ART
Source: PLoS Pathog. 2016 Jul 14;12(7):e1005761. doi: 10.1371/journal.ppat.1005761 (PMC4944956; doi:10.1371/journal.ppat.1005761)
Supplement: S3 Table — (DOCX) [file ppat.1005761.s008.docx]

**S3 Table:** Negative binomial regression models to assess the relationship between 2-LTR circles and Immune Checkpoints expression on CD4^+^ T cells.

| Outcome | Predictor^a^ | Unadjusted | | Adjusted for Current CD4 | | Adjusted for Nadir CD4 | |
| --- | --- | --- | --- | --- | --- | --- | --- |
|  |  | **Result**  **(95%CI) ^c, d^** | **p-value^e^** | **Result**  **(95%CI)** | **p-value** | **Result**  **(95%CI)** | **p-value** |
| 2-LTR circles^b^ | PD1^+^ | 1.20 (0.66 to 2.21) | 0.550 | 1.49 (0.74 to 2.98) | 0.260 | 0.89 (0.57 to 1.39) | 0.590 |
|  | CTLA-4^+^ | 1.38 (0.37 to 5.16) | 0.630 | 1.56 (0.41 to 6.00) | 0.520 | 1.09 (0.41 to 3.09) | 0.880 |
|  | LAG-3^+^ | 1.01 (0.41 to 2.48) | 0.990 | 1.04 (0.43 to 2.54) | 0.930 | 0.84 (0.37 to 1.94) | 0.690 |
|  | TIGIT^+^ | 1.22 (0.49 to 3.03) | 0.670 | 1.32 (0.52 to 3.36) | 0.560 | 1.14 (0.51 to 2.57) | 0.750 |
|  | TIM-3^+^ | 1.35 (0.69 to 2.63) | 0.390 | 1.28 (0.68 to 2.44) | 0.440 | 1.56 (0.86 to 2.83) | 0.140 |
|  | CD160^+^ | 0.59 (0.43 to 0.80) | **0.001** | 0.58 (0.43 to 0.80) | **0.001** | 0.63 (0.47 to 0.86) | **0.003** |
|  | 2B4^+^ | 0.81 (0.54 to 1.21) | 0.310 | 0.82 (0.55 to 1.22) | 0.330 | 0.77 (0.57 to 1.04) | 0.092 |

^a^ Percentage CD4^+^ T cells that express Immune Checkpoint Molecules

^b^ 2-LTR circles units (copies/million CD4^+^ T cells)

^c^ 95% CI = 95% confidence interval

^d^ Result interpretation: fold-change in the outcome (marker of HIV persistence) for each two-fold increase in the predictor (Immune Checkpoint Molecules)

^e^ Statistically significant p values are <0.05 and are bold
