# Supplementary material for: CD4+ T Cells Expressing PD-1, TIGIT and LAG-3 Contribute to HIV Persistence during ART
Source: PLoS Pathog. 2016 Jul 14;12(7):e1005761. doi: 10.1371/journal.ppat.1005761 (PMC4944956; doi:10.1371/journal.ppat.1005761)
Supplement: S4 Table — (DOCX) [file ppat.1005761.s009.docx]

**S4 Table:** Negative binomial regression models to assess the relationship between cell-associated US HIV RNA and Immune Checkpoints expression on CD4^+^ T cells.

| Outcome | Predictor^a^ | Unadjusted | | Adjusted for Current CD4 | | Adjusted for Nadir CD4 | |
| --- | --- | --- | --- | --- | --- | --- | --- |
|  |  | **Result**  **(95%CI) ^c, d^** | **p-value^e^** | **Result**  **(95%CI)** | **p-value** | **Result**  **(95%CI)** | **p-value** |
| US HIV RNA^b^ | PD1^+^ | 1.01 (0.73 to 1.39) | 0.980 | 0.98 (0.64 to 1.51) | 0.940 | 0.96 (0.69 to 1.35) | 0.830 |
|  | CTLA-4^+^ | 1.08 (0.56 to 2.08) | 0.820 | 1.07 (0.55 to 2.07) | 0.840 | 1.12 (0.58 to 2.17) | 0.740 |
|  | LAG-3^+^ | 0.96 (0.57 to 1.61) | 0.870 | 0.95 (0.52 to 1.72) | 0.860 | 0.94 (0.56 to 1.57) | 0.810 |
|  | TIGIT^+^ | 1.10 (0.67 to 1.79) | 0.710 | 1.09 (0.65 to 1.84) | 0.750 | 1.08 (0.67 to 1.74) | 0.760 |
|  | TIM-3^+^ | 1.23 (0.78 to 1.94) | 0.380 | 1.29 (0.80 to 2.08) | 0.300 | 1.24 (0.79 to 1.95) | 0.360 |
|  | CD160^+^ | 0.85 (0.69 to 1.05) | 0.130 | 0.85 (0.69 to 1.05) | 0.120 | 0.86 (0.70 to 1.06) | 0.160 |
|  | 2B4^+^ | 0.95 (0.79 to 1.14) | 0.570 | 0.94 (0.79 to 1.12) | 0.480 | 0.95 (0.79 to 1.13) | 0.560 |

^a^ Percentage CD4^+^ T cells that express Immune Checkpoint Molecules

^b^ US HIV RNA units (copies/million 18S copies)

^c^ 95% CI = 95% confidence interval

^d^ Result interpretation: fold-change in the outcome (marker of HIV persistence) for each two-fold increase in the predictor (Immune Checkpoint Molecules)

^e^ Statistically significant p values are <0.05 and are bold
