# Supplementary material for: CD4+ T Cells Expressing PD-1, TIGIT and LAG-3 Contribute to HIV Persistence during ART
Source: PLoS Pathog. 2016 Jul 14;12(7):e1005761. doi: 10.1371/journal.ppat.1005761 (PMC4944956; doi:10.1371/journal.ppat.1005761)
Supplement: S5 Table — (DOCX) [file ppat.1005761.s010.docx]

**S5 Table:** Negative binomial regression models to compare integrated HIV DNA in cells expressing the Immune Checkpoint Molecule with integrated HIV DNA in cells not expressing the Immune Checkpoint Molecule

| Outcome | Predictor |  |  |
| --- | --- | --- | --- |
|  |  | **Result**  **(95%CI) ^b, c^** | **p-value^d^** |
| Integrated HIV DNA^a^ | Central memory PD-1 | 1.50 (0.94 to 2.40) | 0.093 |
|  | Transitional memory PD-1 | 1.51 (1.00 to 2.29) | **0.053** |
|  | Effector memory PD-1 | 0.67 (0.35 to 1.28) | 0.220 |
|  | Central memory TIGIT | 0.84 (0.59 to 1.20) | 0.330 |
|  | Transitional memory TIGIT | 0.82 (0.51 to 1.30) | 0.387 |
|  | Effector memory TIGIT | 2.71 (1.47 to 5.01) | **0.001** |
|  | Central memory LAG-3 | 1.94 (1.45 to 2.58) | **<0.0001** |
|  | Transitional memory LAG-3 | 1.81 (1.31 to 2.50) | **0.0003** |
|  | Effector memory LAG-3 | 2.50 (1.09 to 5.73) | **0.030** |

^a^ Integrated HIV DNA units (copies/million CD4^+^ T cells)

^b^ 95% CI = 95% confidence interval

^c^ Result interpretation: fold-difference in the outcome (marker of HIV persistence) for cells expressing the IC versus cells not expressing the IC

^d^ Statistically significant p values are <0.05 and are bold
