# Supplementary material for: CD4+ T Cells Expressing PD-1, TIGIT and LAG-3 Contribute to HIV Persistence during ART
Source: PLoS Pathog. 2016 Jul 14;12(7):e1005761. doi: 10.1371/journal.ppat.1005761 (PMC4944956; doi:10.1371/journal.ppat.1005761)
Supplement: S6 Table — (DOCX) [file ppat.1005761.s011.docx]

**S6 Table:** Frequencies of ICs on CD4^+^ T cells in cohort 1 (n=48)

| Percentage ICs | Median | IQR |
| --- | --- | --- |
| PD-1/TIGIT/LAG-3 triple – | 65.8 | 59.0-72.4 |
| PD-1 single + | 4.3 | 2.8-8.0 |
| TIGIT single + | 9.9 | 8.1-12.9 |
| LAG-3 single + | 7.6 | 6.1-10.0 |
| PD-1/TIGIT double + | 4.4 | 2.8-6.6 |
| TIGIT/LAG-3 double + | 1.2 | 0.8-1.8 |
| LAG-3/PD-1 double + | 1.5 | 0.9-2.2 |
| PD-1/TIGIT/LAG-3 triple + | 0.9 | 0.5-1.3 |
